# *In vitro* ruminal fermentation of fenugreek (*Trigonella foenum-graecum* L.) produced less methane than that of alfalfa (*Medicago sativa*)

**DOI:** 10.5713/ajas.20.0114

**Published:** 2020-05-12

**Authors:** Huaxin Niu, Zhongjun Xu, Hee Eun Yang, Tim A McAllister, Surya Acharya, Yuxi Wang

**Affiliations:** 1 College of Animal Science and Technology, Inner Mongolia University for Nationalities, Tongliao, Inner Mongolia 028000, China; 2 Lethbridge Research and Development Centre, Agriculture and Agri-Food Canada (AAFC), Lethbridge, T1J4B1 Alberta, Canada

**Keywords:** Fenugreek, Alfalfa, *In vitro*, Methane, Rumen Bacteria

## Abstract

**Objective:**

The objective of this study was to compare fenugreek (FG) with alfalfa (Alf) in ruminal fermentation and methane (CH_4_) production *in vitro*.

**Methods:**

Whole-plant FG harvested at 11- and 15-wk and Alf harvested at early and mid-bloom maturities, alone or as 50:50 mixture of FG and Alf at the respective maturity, were assessed in a series of 48-h *in vitro* batch culture incubations. Total fermentation gas and methane gas production, dry matter (DM) disappearance, volatile fatty acids, microbial protein and 16S RNA gene copy numbers of total bacteria and methanogens were determined.

**Results:**

Compared to early bloom Alf, FG harvested at 11-wk exhibited higher (p<0.05) *in vitro* DM and neutral detergent fibre disappearance, but this difference was not observed between the mid-bloom Alf and 15-wk FG. Regardless plant maturity, *in vitro* ruminal fermentation of FG produced less (p<0.001) CH_4_ either on DM incubated or on DM disappeared basis than that of Alf during 48-h incubation. *In vitro* ruminal fermentation of FG yielded similar amount of total volatile fatty acids with higher (p<0.05) propionate percentage as compared to fermentation of Alf irrespective of plant maturity. Microbial protein synthesis was greater (p<0.001) with 11-wk FG than early bloom Alf as substrate and 16S RNA gene copies of total bacteria was higher (p<0.01) with 15-wk FG than mid-bloom Alf as substrate. Compared to mid-bloom Alf, 15-wk FG had lower (p<0.05 to 0.001) amount of 16S RNA methanogen gene copies in the whole culture during 48-h incubation.

**Conclusion:**

In comparison to Alf, FG emerges as a high quality forage that can not only improve rumen fermentation *in vitro*, but can also remarkably mitigate CH_4_ emissions likely due to being rich in saponins.

## INTRODUCTION

Methane (CH_4_) production by enteric fermentation in the rumen accounts for 2% to 12% of feed energy loss and is a green-house gas (GHG) that contributes to the global warming potential [1]. Decreasing ruminal CH_4_ production would not only reduce the release of this GHG into the atmosphere, but also possibly offer an avenue to improve feed efficiency in ruminants [2]. Therefore, it is urgently required that nutritionists explore anti-methanogenic substances, preferably through natural feed sources, for enhancing efficiency of nutrient utilization and eco-friendly ruminant production. Naturally occurring plant secondary compounds such as tannins and saponins have been shown to exhibit anti-methanogenic activity depending on source and concentrations [3–5]. Fenugreek (FG, *Trigonella foenum-graecum* L.) has been found to contain steroidal saponins that possess varying biological activities and FG seed and associated extracts have been extensively used as nutraceauticals in human healthcare [6–8].

Fenugreek is an annual legume that has great potential as a forage crop in Western Canada and in other parts of the world because of its sustained quality over the growing season and its drought and frost tolerance [6,9]. Fenugreek especially FG seed contains secondary metabolites including saponins, flavanoids, alkaloids and tannins [7,8,10] that possess varying biological activities [11] and may impact nutrient digestion and metabolism and animal physiology when they are ingested. Researches have shown that FG forage is comparable to alfalfa (Alf) in terms of dry matter (DM) yield and nutrient content [12]. Several studies have been conducted to assess the nutritive value of whole plant FG as forage for cattle [6,9,13]. However, there is no information available on the effects of FG on ruminal CH_4_ production. The objective of this study was to compare the *in vitro* ruminal fermentation of FG and Alf with emphasis on its impact on CH_4_ production.

## MATERIALS AND METHODS

### Substrate and inoculum

Whole FG (*Trigonella foenum-graecum*; Tristar FG) plants at 11-and 15-wk after sowing and Alf (*Medicago sativa*; AC Grazeland Br) at early bloom and mid bloom stages of the regrowth were cut above ground from three separate plots of the Swinton silt loam soil for each forage at the Lethbridge Research and Development Centre, AB, Canada. The seeding rate for FG was 15 kg/ha and soil was amend to 45.4 kg available N at seeding. Edge pre-emergence herbicide was applied prior to seeding and Odyssey herbicide was applied during the growing season to control volunteer weeds. The Alf plots had been established for three years and have been used for hay production in previous years. The maturity of whole-plant FG harvested at 11 and 15 wk after sowing corresponded to that of early- and mid-bloom Alf. Forage samples from the three plots were combined, freeze-dried and ground to pass through a 1-mm screen.

*In vitro* incubations were conducted to compare the two forages at the two different maturities, with FG harvested at 11 wk comparing to Alf harvested at early bloom and a FG and Alf mixture at 50:50 ratio (DM basis; FA), and FG harvested at 15 wk comparing to Alf harvested at mid-bloom and the corresponding 50:50 mixture (FA). Comparison between different maturities of the same forage was excluded because the focus of this study was to compare two forages.

The same three ruminally cannulated Angus heifers (550± 50 kg body weight) were used as rumen fluid donors for all incubations. The heifers were fed (DM basis) a forage diet containing 50% Alf hay, 35% barley silage, 12% dry-rolled barley, and 3% of a vitamin and mineral supplement as per the National Research Council [14]. All heifers were fed at 08:00 h and provided *ad libitum* access to feed and water and were cared for in accordance with standards of Canadian Council on Animal Care [15]. Rumen fluid was collected 2 h after the morning feeding from five locations within the rumen, strained through four layers of cheesecloth, combined in equal volumes among three cattle and immediately transported in an anaerobic and pre-warmed container to the laboratory. Rumen fluid was then combined (1:2, v/v) with pre-warmed (39°C) mineral buffer [16] to generate the inoculum. The inoculum used for comparing FG harvested at 11 wk to Alf harvested at early bloom was modified by replacing 1.0 g/L of ammonium bicarbonate with equal amount of ^15^N-enriched ammonium sulphate (Sigma Chemical Co, St Louis, MO, USA) to be used as microbial N marker.

### *In vitro* incubations and measurements

Incubation was conducted in 125-mL serum bottles with the preloaded (0.5 g) substrate of FG, Alf, or FA. Pre-warmed inoculum was dispensed under a stream of O_2_-free CO_2_ into pre-warmed serum vials (60 mL/vial), immediately sealed and affixed to a rotary shaker platform (140 rpm) in an incubator at 39°C. Vials containing no substrate were also inoculated and incubated as blank controls. Vials for 0 h incubation were immediately placed into ice-water bath after inoculation. Incubation was repeated twice (2 runs) for the forages at early maturity and thrice for forages at later maturity (3 runs).

Gas production (GP) from each vial was measured after 6, 12, 24, and 48 h of incubation using a water displacement device as described by Wang et al [17]. A 15-mL gas sample was collected from each vial prior to gas measurement to determine CH_4_ concentration [18]. After gas measurement, three vials from each substrate and blank controls at each time point were placed on ice and processed to estimate DM disappearance (DMD), volatile fatty acid (VFA) and ammonia [3]. For the early maturing forages microbial N production was estimated using ^15^N as marker [3]. For mature forages, sub-samples (2.0 mL) of whole culture was collected from each vial for DNA extraction in order to estimate total bacteria and methanogens based on 16S rRNA and mcrA gene copies respectively.

### DNA extraction and real-time quantitative polymerase chain reaction

Sample of whole cultures were lyophilized and ball ground using a planetary micro mill (30 1/S, 3 min, 10-mm steel balls, Retsch Inc., Newtown, PA, USA) and then metagenomic DNA was extracted using the Qiagen OIAamp DNA stool mini kit (Qiagen Inc., Valencia, CA, USA) as recommended by the manufacture with the exception that a bead-beating step (5 min, maximum speed) using a Bead Ruptor 24 Elite (Omni International Inc., Kennesaw, GA, USA) was added. Nucleic acids were precipitated with ammonium acetate and followed by isopropanol, washed twice with ethanol and suspended in Tris-ethylenediaminetetraacetic acid buffer. The quality of the DNA was assessed via electrophoresis on 1.2% agarose gel (w/v), and the DNA concentration of each sample was determined using a Nanodrop 2000 (Thermo Fisher Scientific Inc., Madison, WI, USA). The DNA samples were stored at −40°C until analyzed.

The polymerase chain reaction (PCR) primers used for real-time quantitative PCR (qPCR) quantification of total bacteria and methanogens 16S rRNA and mcrA sequences are described in Table 1. Real-time qPCR amplification was performed on a Bio-Rad CFX 96 Connect Real-Time PCR Detection System (Bio-Rad Laboratories Inc., Hercules, CA, USA), and data were analyzed using Bio-Rad CFX Manager software (version 3.0). Standard curves were generated using 10-fold serial dilutions of DNA plasmid standards containing the target gene sequences of the respective microbial groups. Negative controls were included in the analyses (H_2_O instead of DNA). The PCR cycling conditions were an initial denaturation step at 95°C for 3 min, followed by 40 cycles of denaturation at 95°C for 25 s, annealing at 50°C for 30 s, and extension at 72°C for 45 s. A melting curve analysis was performed by slowly cooling the reaction mixture from 95°C to 65°C to detect nonspecific amplification products. The copy number of marker genes of the total bacteria and methanogens were expressed per ml of whole culture.

### Chemical analysis

The DM was determined by drying samples at 105°C for 16 h in a forced-air oven (AOAC, # 930.15) [19] and organic matter was determined by ashing in a muffle furnace (AOAC, # 943.01) [19]. The samples were ball ground in a planetary micro mill (Retsch Inc., USA) and analyzed for total N estimation by flash combustion analysis using a NA1500 nitrogen analyzer (Carlo Erba Instruments, MI, Italy). Neutral detergent fibre (NDF) and acid detergent fibre (ADF) were performed using an Ankom 200 system (Ankom Technology Corp., Fairport, NY, USA), with addition of sodium sulfite and alpha-amylase for NDF but without for ADF analysis as described by McGinn et al [20]. Content of steroidal saponins from FG and Alf was determined using method described by Wang et al [23] with smilagenin (Sigma, USA) as a standard.

### Calculations and statistics analysis

Microbial N production was calculated as described by Wang et al [3]. The *in vitro* NDF disappearance (NDFD) after 48-h incubation was calculated as the difference of NDF in the substrate before and after 48-h incubation. Calculations were calibrated by blank control.

The data were statistically analyzed using the Mixed pro cedure of SAS (SAS Institute Inc., Cary, NC, USA) as a randomized complete block design with treatment as the fixed effect, run as block and vials as statistical unit. The model used for analysis of time-course (repeated measures) data included time and the time×treatment interaction. Different variance and covariance assumption structures were initially test in the model and the type with lowest Akaike information criterion value was used in the final analysis. When these effects (time or time×treatment interaction) were significant (i.e. p<0.05), means of the treatments were compared at each time point. Differences between treatment means was determined by LSMEANS with the PDIFF option in SAS and declared significant at p<0.05.

## RESULTS

Fenugreek harvested at 11 wk had numerically higher concentration of crude protein (CP), but numerically lower concentrations of NDF and ADF than that harvested at 15 wk (Table 2). A similar trend was also observed for Alf harvested between early and mid-bloom stages. For substrates used in each comparison, FG had numerically lower concentrations of NDF and ADF but similar CP content as compared to Alf. The concentration of steroidal sapongenin in 11- and 15-wk FG were 113.2 and 95.8 mg smilagenin equivalent/100 g DM respectively, but was not detectable in Alf.

Significant time effect (p <0.05) on fermentation products was observed and therefore data were presented at each time points. Fenugreek harvested at 11-wk had higher *in vitro* DMD at 6, 12, 24, and 48-h incubation (p<0.05 to 0.001) than Alf harvested at early bloom (Table 3). The FA also had higher *in vitro* DMD than Alf at 6- and 48-h incubation. *In vitro* NDFD at 48-h incubation was higher (p<0.05) for FG than for Alf and FA. On the contrary, GP (mL/g DM) from incubation of Alf was greater at 6, 12, 24, and 48-h incubation (p<0.05 to 0.001) than that of FG. CH_4_ production from 11 wk FG per g DM incubated was 15% to 33% lower (p<0.001) than for early bloom Alf after 6, 12, 24, and 48 h of incubation. The difference of CH_4_ production between *in vitro* ruminal fermentation of FG and Alf at each incubation time point (23% to 62%) was even greater when CH_4_ production was expressed on per g DM digested basis. CH_4_ production from *in vitro* ruminal fermentation of FA at each incubation time was intermediate and was higher (p<0.01) than that of FG, but lower (p<0.01) than that of Alf either on the basis of DM incubated or DM digested.

With mature forages, no difference was found in *in vitro* DMD, 48-h NDFD and GP between FG harvested at 15 wk and Alf harvested at mid-bloom. *In vitro* DMD of FG at 24-h incubation was lower (p<0.05) than for Alf and FA (Table 4). In contrast, similar to that observed in FG harvested at 11 wk and Alf harvested at early bloom, CH_4_ production from *in vitro* ruminal fermentation of FG at 6 to 48 h was lower (p< 0.01 to 0.001) than that from Alf with FA being intermediate. This difference was the same irrespective of expressing CH_4_ production on the basis of DM incubated or DM digested.

Fermentation of Alf harvested at early bloom stage yield ed higher (p<0.01) ammonia concentration than 11-wk FG and FA at 6 h of incubation, but there was no difference in ammonia concentration among the three substrates between 12 and 48 h of incubation (Table 5). Similarly, there was no difference in total VFA concentration and molar percentage of acetate throughout the 48-h incubation. In contrast, molar percentage of propionate was higher (p<0.05 to 0.001), but molar percentage of butyrate was lower (p<0.01) for FG than Alf. Acetate:propionate ratio in VFA produced during 48-h fermentation of FG was lower (p<0.05 to 0.001) than Alf and the same difference was also observed between FA and Alf at 12, 24, and 48 h of the incubation.

With mature forages, ammonia concentration in the liq uid fraction of the whole culture at 12 and 24-h incubation was higher (p<0.01) for Alf than for FG (Table 6). However, this difference was not observed at 6 and 48 h of the incubation. Consistent with finding in FG harvested at 11 wk and Alf harvested at early bloom, VFA concentrations were similar among three substrates regardless of the incubation time. Molar percentage of propionate ranked FG>FA>Alf (p<0.001), whereas molar percentages of butyrate and branch-chain VFA ranked Alf>FA>FG (p<0.05 to 0.001) for all incubation times. There was no difference in total VFA concentration and molar percentage of acetate throughout the 48-h incubation. Acetate: propionate ratio in VFA produced during 48-h fermentation was also ranked as Alf>FA>FG (p<0.001).

**Figure 1:**
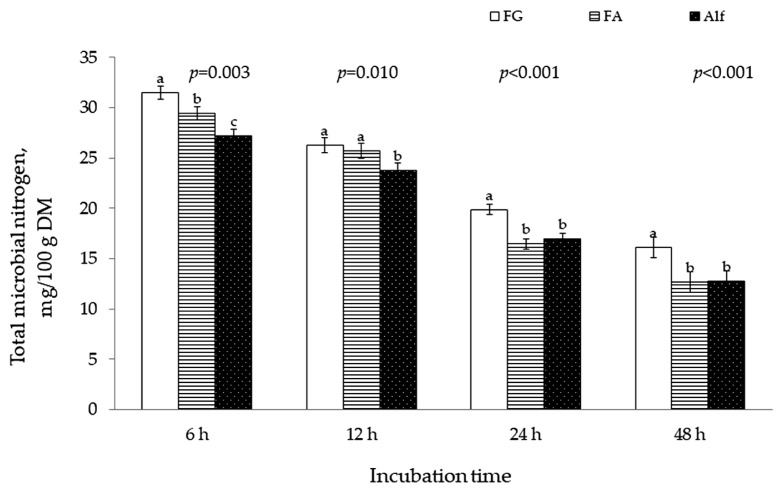
Microbial N production of fenugreek (FG) harvested at 11 wk, alfalfa (Alf) harvested at early bloom and their 50:50 mixture (FA) during 48-h *in vitro* ruminal incubation.

Microbial N production was greater (p <0.05) for 11-wk FG than for early bloom Alf at 6, 12, 24, and 48-h incubation (Figure 1). There was no difference between FA and Alf in microbial N production at 24 and 48-h incubation although incubation of FA produced more (p<0.05) microbial N than Alf at 6 and 12 h of the incubation.

16S rRNA copy numbers associated with total bacteria in the whole culture was greater at 12, 24, and 48 h of the incubation (p<0.05 to 0.01) for 15-wk FG than for mid-bloom Alf as substrate with FA being intermediate (Table 7). On the contrary, the amount of mcrA gene copies in the whole culture was greater (p<0.05 to 0.001) for Alf than for FG at 6, 12, and 24-h incubation and was slightly greater (p = 0.093) for Alf than for FG at 48-h incubation.

## DISCUSSION

There is little information on comparison of ruminal fermentation of FG and Alf forage. Mir et al [6] reported that 15- and 19-wk green-house grown FG had greater *in vitro* DMD than that of early bloom Alf but with similar VFA production. Mustafa et al [24] showed that 15-wk FG hay had higher ruminal degradabilities of DM, CP, and ADF and higher *in vitro* DMD compared to the full bloom regrowth Alf. The present study showed that GP for early bloom Alf was higher than those for 11-wk FG, which is consistent with the results of Mir et al [6], but no difference was found in GP from *in vitro* ruminal incubation of 15-wk FG and mid-bloom Alf, which is in agreement with Farivar et al [25]. The discrepancy in GP among studies comparing FG and Alf could be attributed to different maturities of the forages used in these studies. Nevertheless, the observations of greater *in vitro* DMD but less GP with 11-wk FG than with early bloom Alf indicated that *in vitro* ruminal fermentation of 11-wk FG was more efficient towards to producing microbial protein than that of early bloom Alf because total VFA production was similar between the two forages. This is supported by the observations that microbial protein synthesis was greater for *in vitro* fermentation of 11-wk FG than for early bloom Alf and that total bacteria populations was higher for *in vitro* fermentation of 15-wk FG than for mid-bloom Alf in this study.

Incubation of FG led to greater microbial N in FG har vested at 11 wk than Alf harvested at early bloom was consistent with greater total bacterial population in FG harvested at 15 wk and Alf harvested at mid-bloom. This suggests, regardless of the plant maturity, that fermentation of FG would yield greater microbial protein than fermentation of Alf. This is likely due to the numerically lower NDF and ADF but slightly higher N in FG than in Alf, as well as the presence of plant secondary compounds such as steroidal saponin in FG. Antimicrobial and anti-protozoal activity of steroidal saponin are well recognized, which are bacterial species specific, mainly inhibiting G^+^ positive bacteria. However, Wang et al [3] showed dose-dependent response of rumen microbial protein synthesis to steroidal saponin from *Yucca schidigera* (*Y. schidigera*), with the concentration of 15 μg/mL increasing microbial protein synthesis and higher than that decreasing microbial production. Estimated steroidal saponin (from FG) concentration in the whole culture of this study ranged 10 to 12 μg/mL. At these concentrations, FG steroidal saponin may also have promoted microbial protein synthesis. Dey et al [26] also showed saponin extracts from FG leaves increased microbial protein synthesis.

The most interesting finding of the study was that ruminal fermentation of FG produced less CH_4_ than fermentation of Alf and this was consistent across two maturities of the two forages and expressed either on per unit of DM incubated or on the basis of DM disappeared. It is commonly accepted that feedstuffs which have higher GP and *in vitro* DMD tend to have higher CH_4_ production per gram DM incubated [4, 26,27]. The observation of less methane production of FG than that of Alf despite the discrepancies in DMD and GP per unit of substrate between the two forages at two maturities indicated FG may contain specific anti-methanogenic activity. This is supported by the observation of lower methanogen gene copies with FG than with Alf. Several biological compounds such as steroidal saponins and phenolic compounds that possess varying biological activities have been found in FG [11,12,28]. Among them, steroidal saponins have been reported to possess anti-methanogenic activity in both *in vitro* or *in vivo* studies [4,29,30]. In this study, steroidal saponin was found in FG but not in Alf. Other studies also reported that FG and its seed extracts are rich in saponins [6,8,12]. Therefore, compared to Alf it is likely that steroidal saponins in FG contributed to its lower methane production during ruminal fermentation. It needs to be pointed out that this study used whole-plant FG and Alf as substrates and therefore was unable to identified exactly what factor or factors in FG lead to the decreased methane production compared to Alf. Further research is needed to elucidate the mechanism by which ruminal fermentation of FG yielded lower methane production than that fermentation of Alf.

The lower methane production was accompanied by higher propionate and lower acetate: propionate ratio in VFA for *in vitro* ruminal fermentation of FG than that of Alf in this study. Propionate is generally regarded as an alternative metabolic H_2_ sink to methane during rumen fermentation. This suggests that either fermentation of FG enhanced propionate production thereby decreased H_2_ sink to methane formation, or decreased methane production through decreasing methanogenic activity and thereby increased H_2_ as a sink to propionate. Studies have shown that plants rich in saponins selectively modulated the rumen microbial populations resulting in an improvement of rumen fermentation towards enhanced propionate and decreased CH_4_ production [31,32]. Our previous studies demonstrated that steroidal saponin from *Y. schidigera* selectively decreased ruminal cellulolytic bacteria activity but increased *Selenomonas ruminantium*, a propionate-producing bacteria, resulting in decreased acetate: propionate ratio [33]. Meta-analyses also showed that saponins or saponin containing plants increased propionate concentrations [7] and that decreased CH_4_ production was accompanied by reduced acetate:propionate [32]. In addition, rumen enteric microbiome were modified when plants rich in saponins were fed to ruminant livestock, resulting in increased propionate, and decreased methane production [33,34]. Therefore, it is likely that the presence of steroidal saponin in FG but not in Alf in this study contributed to the increased propionate production during ruminal fermentation of FG.

Jayanegara et al [4] summarized *in vitro* studies on effects of saponins from *Y. schidigera*, Quillaja and tea on ruminal fermentation and found that steroidal saponins from all of these sources decreased methane production and acetate: propionate ratio with no or little effects on total GP or DMD. These results as well as the work of others [29] suggest that steroidal saponin from FG may have similar effects on ruminal fermentation to other reported steroidal saponin. Further research is needed to define the role of FG saponins in modifying ruminal fermentation in particular its effects on rumen microbial activity, VFA profile and methane production.

The difference of ammonia concentration in fermentation liquid fraction between two forages is likely attributable to the differences in N and plant secondary contents. It has been shown that ammonia accumulation in closed *in vitro* system increased as the substrate N increasing [35]. Both FG and Alf contained numerically higher N at early than late maturity. At early maturity, FG also contained numerically higher N than Alf. These may partially explain the higher ammonia concentration resulted from fermentation of early mature than late mature FG and Alf and from 11-wk FG than early bloom Alf. However, the inconsistent difference in ammonia concentration between FG and Alf in different mature stages may indicate effects of plant secondary compounds in FG on N metabolism in the rumen was not as obvious as that observed on methane production. A common observation of plant polyphenolic compounds effects on rumen fermentation is the decrease of ammonia concentration [36]. Previous researches have showed that *Y. schidigera*, steroidal saponins decreased ruminal ammonia concentration [3], but this was not observed for Quillaja and tea saponins [4]. Nevertheless, the lower ammonia concentration was accompanied with lower branch-chain VFA concentration for fermentation of 15-wk FG than for that of mid bloom Alf. This coupling with the similar N content of the two forages at mature stage indicated that protein degradation might have been decreased by FG plant secondary compounds in this study.

## CONCLUSION

Fenugreek harvested at 11wk had higher *in vitro* ruminal fermentation than early bloom Alf. However, regardless of plant maturity, *in vitro* ruminal fermentation of FG produced less methane than fermentation of Alf either on DM incubated or on DM digested basis, partially due to the presence of steroidal saponins in FG. Fermentation of FG also produced VFA with higher propionate proportion and lower acetate:propionate ratio and yielded greater amount of microbial protein or larger bacterial population than Alf. Therefore, whole plant FG could be an alternative legume forage for ruminants, in particular in mitigation of enteric methane emission from the cattle industry. Animal studies need to be conducted to confirm its *in vivo* effects.

## Figures and Tables

**Table 1:** Primers for real-time quantitative polymerase chain reaction

Target	Primer	Primer sequence (5′–3′)	Amplicon size (bp)	Annealing temperature (°C)	Reference
Total bacteria (16S)	F	CTCCTACGGGAGGCAGCAGT	156	60	[21]
	R	TTACCGCGGCTGCTGGCAC			
Methanogens (mcrA)	F	TTCGGTGGATCDCARAGRGC	140	58	[22]
	R	GBARGTCGWAWCCGTAGAATCC			

**Table 2:** Chemical composition (mg/g dry matter) of the forages

Forages	Maturity	OM	CP	NDF	ADF	Steroidal saponin1)
Fenugreek	11 wk	890.8±9.93	235.5±1.67	264.2±6.56	229.3±2.16	113.2±7.34
	15 wk	890.0±2.18	177.5±1.25	377.9±5.31	344.4±2.21	95.8±3.24
Alfalfa	Early bloom	897.1±6.58	211.6±1.72	331.4±7.38	279.9±3.69	ND
	Mid bloom	898.3±7.18	173.7±2.51	438.9±7.12	371.1±4.13	ND

Mean±standard deviation, n = 4.

OM, organic matter; CP, crude protein; NDF, neutral detergent fibre; ADF, acid detergent fibre; ND, not detected.

1) mg smilagenin equivalent/100 g.

**Table 3:** Dry matter disappearance (DMD), neutral detergent fibre disappearance (NDFD), gas production (GP) and methane production during 48-h *in vitro* ruminal incubation of forages in the early growth

Item	Incubation time (h)	FG	FA	Alf	SEM	p-value
DMD (%)	6	35.4^a^	32.6^a^	29.2^b^	1.65	0.004
	12	50.7^a^	47.1^ab^	43.1^b^	2.01	0.048
	24	60.2^a^	55.7^b^	53.8^b^	3.25	0.002
	48	68.9^a^	67.9^a^	65.2^b^	0.71	<0.001
NDFD (%)	48	44.3^a^	40.7^b^	40.9^b^	0.81	0.016
GP (mL/g DM)	6	95.5^c^	100.6^b^	103.5^a^	8.79	<0.001
	12	165.8^b^	169.0^b^	172.4^a^	3.57	<0.001
	24	190.9^b^	195.2^ab^	197.5^a^	2.74	<0.001
	48	202.3^b^	213.4^a^	212.9^a^	2.69	0.012
CH_4_ (mL/g DM)	6	12.0^c^	13.6^b^	16.0^a^	1.59	<0.001
	12	20.4^c^	22.4^b^	26.5^a^	4.27	<0.001
	24	25.4^c^	30.0^b^	34.3^a^	3.08	<0.001
	48	32.8^c^	35.9^b^	38.3^a^	2.91	<0.001
CH_4_ (mL/g DMD)	6	34.2^c^	42.5^b^	55.3^a^	6.63	<0.001
	12	41.4^c^	48.2^b^	62.1^a^	12.27	<0.001
	24	42.8^c^	54.6^b^	64.1^a^	8.67	<0.001
	48	47.7^c^	52.8^b^	58.8^a^	4.81	<0.001

DMD, Dry matter disappearance; NDFD, neutral detergent fibre disappearance; GP, gas production; CH_4_, methane FG, fenugreek; FA, mixture of fenugreek and alfalfa at the ratio of 50:50; Alf, alfalfa; SEM, standard error of the mean.

a–c Means within a row with different lowercased letters differ (p<0.05).

**Table 4:** Dry matter disappearance (DMD), neutral detergent fibre disappearance (NDFD), gas production (GP), and methane (CH_4_) production during 48-h *in vitro* ruminal incubation of mature forages

Item	Incubation time (h)	FG	FA	Alf	SEM	p-value
DMD (%)	6	34.0	35.1	35.0	1.23	0.124
	12	49.1	48.2	48.0	0.96	0.102
	24	52.4^b^	55.5^a^	54.8^a^	2.00	0.013
	48	67.4	67.9	66.3	0.64	0.086
NDFD (%)	48	40.5	41.4	40.3	0.96	0.668
GP (mL/g DM)	6	93.8	95.6	94.1	1.69	0.105
	12	139.6	141.9	139.3	1.47	0.294
	24	177.6	180.0	176.7	1.95	0.305
	48	196.3	203.7	201.1	4.12	0.438
CH_4_ (mL/g DM)	6	14.1^c^	16.1^b^	18.5^a^	2.06	<0.001
	12	21.1^c^	25.3^b^	27.0^a^	1.87	<0.001
	24	32.2^b^	38.2^a^	38.7^a^	2.88	<0.001
	48	42.2^b^	45.7^a^	47.8^a^	1.60	0.002
CH_4_ (mL/g DMD)	6	42.1^b^	46.9^a^	48.8^a^	4.51	<0.001
	12	47.1^c^	52.7^b^	56.3^a^	3.42	<0.001
	24	61.1^b^	68.9^a^	70.2^a^	3.71	0.017
	48	62.7^c^	64.5^b^	69.2^a^	2.73	<0.001

DMD, dry matter disappearance; NDFD, neutral detergent fibre disappearance; GP, gas production; CH_4_, methane; FG, fenugreek; FA, mixture of fenugreek and alfalfa at the ratio of 50:50; Alf, alfalfa; SEM, standard error of the mean.

a–c Means within a row with different lowercased letters differ (p<0.05).

**Table 5:** Ammonia concentration and profile of volatile fatty acids (VFA) in liquid fraction of whole culture of *in vitro* ruminal incubation of forages in the early growth

Item	Incubation time (h)	FG	FA	Alf	SEM	p-value
Ammonia (mmol/L)	6	17.4^a^	17.0^a^	15.2^b^	0.36	0.004
	12	20.7	20.8	19.5	1.88	0.166
	24	27.3	28.0	26.3	0.68	0.189
	48	34.6	35.4	34.1	1.58	0.110
Total VFA (mmol/L)	6	60.4	56.6	60.4	2.44	0.239
	12	73.5	68.7	71.2	2.83	0.474
	24	82.6	85.1	87.9	2.67	0.346
	48	83.8	94.7	94.4	3.76	0.095
Acetate (%)	6	68.0	67.4	67.8	0.52	0.358
	12	67.2	66.5	66.9	0.58	0.176
	24	65.8	65.3	65.8	0.74	0.086
	48	64.7	65.1	65.9	0.55	0.114
Propionate (%)	6	19.9^a^	18.9^b^	18.8^b^	0.19	0.001
	12	19.0^a^	18.5^ab^	18.2^b^	0.10	<0.001
	24	19.0^a^	18.4^ab^	18.1^b^	0.26	0.013
	48	18.5^a^	18.1^a^	17.5^b^	0.18	0.007
Butyrate (%)	6	8.0^b^	9.0^a^	9.0^a^	0.47	0.007
	12	8.3^b^	9.4^a^	9.8^a^	0.41	0.002
	24	8.9^b^	9.7^a^	9.9^a^	0.27	0.003
	48	9.3	9.6	9.8	0.36	0.711
Branch chain VFA (%)	6	2.9	2.9	2.9	0.20	0.932
	12	4.7^a^	4.5^a^	4.0^b^	0.11	0.001
	24	6.1	6.2	6.4	0.32	0.841
	48	7.0	7.4	7.3	0.31	0.587
Acetate:propionate	6	3.43^b^	3.56^a^	3.60^a^	0.023	0.004
	12	3.54^b^	3.59^b^	3.67^a^	0.031	0.011
	24	3.47^b^	3.54^b^	3.64^a^	0.096	0.018
	48	3.50^b^	3.59^b^	3.76^a^	0.067	0.046

SEM, standard error of the mean.

VFA, volatile fatty acids; FG, fenugreek; FA, mixture of fenugreek and alfalfa at the ratio of 50:50; Alf, alfalfa; SEM, standard error of the mean.

a,b Means within a row with different lowercased letters differ (p<0.05).

**Table 6:** Ammonia concentration and profile of volatile fatty acids (VFA) in liquid fraction of whole culture of *in vitro* ruminal incubation of mature forages

Item	Incubation time (h)	FG	FA	Alf	SEM	p-value
Ammonia (mmol/L)	6	11.6	11.9	12.1	0.52	0.458
	12	14.4^b^	16.6^a^	17.2^a^	0.39	<0.001
	24	22.6^c^	23.9^b^	25.2^a^	0.72	0.004
	48	29.6	30.3	30.2	1.26	0.591
Total VFA (mmol/L)	6	53.5	51.6	51.7	2.36	0.124
	12	63.6	61.7	61.0	2.16	0.327
	24	74.2	74.0	73.8	1.67	0.975
	48	85.3	82.3	79.4	3.00	0.134
Acetate (%)	6	67.9^b^	68.1^b^	68.6^a^	0.28	0.002
	12	66.7	66.8	67.1	0.23	0.186
	24	65.2	65.3	65.7	0.14	0.060
	48	64.8^b^	65.1^ab^	65.4^a^	0.21	0.056
Propionate (%)	6	20.5^a^	19.6^b^	18.5^c^	0.47	<0.001
	12	20.2^a^	19.5^b^	18.5^c^	0.57	<0.001
	24	20.1^a^	19.2^b^	18.5^c^	0.58	<0.001
	48	19.6^a^	18.7^b^	17.9^c^	0.53	<0.001
Butyrate (%)	6	8.0^b^	8.3^ab^	8.7^a^	0.59	0.008
	12	8.7^c^	9.1^b^	9.5^a^	0.62	<0.001
	24	9.3^c^	9.7^b^	9.9^a^	0.50	<0.001
	48	9.4^c^	9.7^b^	9.8^a^	0.51	<0.001
Branch-chain VFA (%)	6	3.7^b^	3.9^ab^	4.0^a^	0.15	0.040
	12	4.2^c^	4.5^b^	4.8^a^	0.16	<0.001
	24	5.2^c^	5.6^b^	5.8^a^	0.11	<0.001
	48	6.0^c^	6.4^b^	6.7^a^	0.15	<0.001
Acetate:propionate	6	3.32^c^	3.48^b^	3.71^a^	0.068	<0.001
	12	3.30^c^	3.43^b^	3.63^a^	0.087	<0.001
	24	3.25^c^	3.40^b^	3.56^a^	0.097	<0.001
	48	3.32^c^	3.45^b^	3.65^a^	0.085	<0.001

VFA, volatile fatty acids; FG, fenugreek; Alf, alfalfa; FA, mixture of fenugreek and alfalfa at the ratio of 50:50; SEM, standard error of the mean.

a–c Means within a row with different lowercased letters differ (p<0.05).

**Table 7:** 16S rRNA gene copy numbers of total bacteria and methanogen archaea in the whole culture of *in vitro* ruminal incubation of mature forages

Item	Incubation time (h)	FG	FA	Alf	SEM	p-value
Total bacteria (×10^8^/mL)	6	9.62	8.64	7.81	1.696	0.185
	12	9.84^a^	7.72^b^	6.47^b^	0.960	0.007
	24	6.61^a^	4.86^b^	4.68^b^	0.552	0.042
	48	2.50^a^	2.68^a^	1.77^b^	0.282	0.023
Methanogen (×10^4^/mL)	6	1.44^b^	1.84^ab^	1.90^a^	0.795	0.042
	12	1.94^b^	1.61^ab^	2.25^a^	0.784	0.036
	24	2.10^c^	3.02^b^	4.23^a^	0.533	<0.001
	48	2.26	2.63	2.94	0.452	0.093

FG, fenugreek; Alf, alfalfa; FA, mixture of fenugreek and alfalfa at the ratio of 50:50; SEM, standard error of the mean.

a,b Means within a row with different lowercased letters differ (p<0.05).
